# A review of pollution-based real-time modelling and control for sewage systems

**DOI:** 10.1016/j.heliyon.2024.e31831

**Published:** 2024-05-31

**Authors:** Rodrigo da Silva Gesser, Holger Voos, Alex Cornelissen, Georges Schutz

**Affiliations:** a University of Luxembourg, 29 Av. John F. Kennedy, Luxembourg City, 1855, Luxembourg; b RTC4Water, 62a Grand-Rue, Roeser, 3394, Luxembourg

**Keywords:** Real-time control, Water quality, Sewage systems

## Abstract

Conventional solutions for wastewater collection focus on reducing overflow events in the sewage network, which can be achieved by adapting sewer infrastructure or, a more cost-effective alternative, by implementing a non-engineering management solution. The state-of-the-art solution is centered on Real-Time Control (RTC), which is already resulting in a positive impact on the environment by decreasing the volume of wastewater being discharged into receiving waters. Researchers have been continuing efforts towards upgrading RTC solutions for sewage systems and a new approach, although rudimentary, was introduced in 1997, known as Pollution-based RTC (P-RTC), which added water quality (concentration or load) information explicitly within the RTC algorithm. Formally, P-RTC is encompassed of several control methodologies using a measurement or estimation of the concentration (i.e. COD or ammonia) of the sewage throughout the network. The use of P-RTC can result in a better control performance with a reduction in concentration of overflowing wastewater observed associated with an increase of concentration of sewage arriving at the Wastewater Treatment Plant (WWTP). The literature revealed that P-RTC can be differentiated by: (1) implementation method; (2) how water quality is incorporated, and (3) overall control objectives. Additionally, this paper evaluates the hydrological models used for P-RTC. The objective of this paper is to compile relevant research in pollution-based modelling and real-time control of sewage systems, explaining the general concepts within each P-RTC category and their differences.

## 1 Introduction

Modernisation increased the use of many fundamental resources and thus required an evolution of the techniques responsible for handling, distributing, collecting and disposing of these resources. The prime example is water, which is arguable the most important available resource, and wastewater, both having critical environmental, social and economical impacts [7]. In most countries, water is mainly collected, treated and distributed for personal use in dwellings, while wastewater is the result of its consumption: wastewater, is transported by the sewage system, treated in Waste Water Treatment Plants (WWTPs), and discharged into receiving water bodies such as river or seas [71]. Wastewater, the focus of this paper, needs to be treated to avoid negative impacts on the environment and, therefore, requires advanced techniques to guarantee that these resources are being efficiently and reliably collected [48].

The main issue in sewage systems is the occurrence of overflows in a combined network, characterised by the combination of rainwater and sewage that are collected and transported through the wastewater system. Here, Combined Sewer Overflow (CSO) are caused by rainwater that flows into the sewage system during rain events and overloads the sewers due to insufficient storage [62]. Increases in CSO events may be caused by climate change, increases in urban population and reduction in soil rainwater retention [32]. For completeness, the papers [60] and [46] details the state of the art for overflow prediction in wastewater systems.

Therefore, minimising CSO events is of utmost importance for every urban area [62]. This can be achieved by increasing storage volume, resizing pipes and pumps or removing rainwater from the sewage system by adding Sustainable Urban Drainage Systems (SUDS). However, a more cost-effective solution is to implement software-based approaches. A few of such solutions have been implemented in actual applications, such as the study from [13] implementing a Model Predictive Control (MPC) strategy in Barcelona. [61] developed a global optimal control solution in Quebec. [25] and [26] implemented a multi-objective MPC approach in Luxembourg and [51] designed a rule-based algorithm to control a sewer system in Copenhagen.

**Figure 1:**
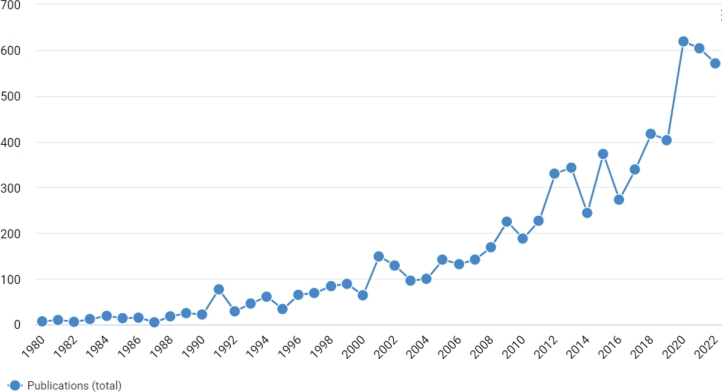
Total publications per year from 1980 to 2022, estimated by searching the key words “Real time control” and “Wastewater” in the data-set provided by [34].

Real-Time Control (RTC) is a common approach for global control of wastewater networks. The underlying reason for applying a RTC is that wastewater networks are systems with a highly dynamic behaviour, with constantly changing operational variables, necessitating a controller capable of reacting in real time [20]. The overall literature of RTC applied to drainage systems is growing constantly, as can be seen in Fig. 1 and according to the online data-set available at [34], the estimation of published studies on the topic is approximately 7000 papers. There are several reviews, detailing many different algorithms and implementations, for example: [32], [73] and [95] reviewed modelling and real-time control of sewage systems, in the research of [16] the performance of different RTC methods is evaluated and [96] illustrated the impact of RTC algorithms on storm-water control measures.

However, the usual application of RTC does not implicitly consider the pollution load [80], which is only feasible by either using more complex models or by adding water quality information to the algorithm. Measuring water quality in the sewage system is difficult because of the harsh environment and associated maintenance costs [19]. Incorporating quality data results in the possibility to explicitly influence the sewage network and manipulate the system to ensure better performance, which is possible, but not guaranteed using a purely volume-based approach. Improvements derived from using water quality data in RTC can include reducing overflow with highly concentrated wastewater and increasing load being directed to the WWTP.

Therefore, efforts have been made to improve RTC using pollution-based approaches as seen in the works of [48], [78] and [85]. Also, studies in water quality prediction and assessment in sewer systems are available [39], [57], [99].

Although in the literature there are reviews on water quality modelling [100], water quality dynamics and in-sewer processes [31], a search through SCOPUS and Google Scholar revealed a lack of studies reviewing specifically Pollution-based RTC (P-RTC).

Therefore, the aim of this paper is to provide a review of P-RTC, focusing on the models used to describe pollution in SUDS for control purposes and related control techniques that have been developed so far. It is imperative to provide a comprehensive guide for researchers and practitioners about the substantial impact of applying RTC with explicit quality objectives. Finally, the paper discusses what new research could result from the identified literature gaps and how filling these gaps might influence the future of sewage systems control and modelling.

## 2 Modelling of sewage systems for control

**Figure 2:**
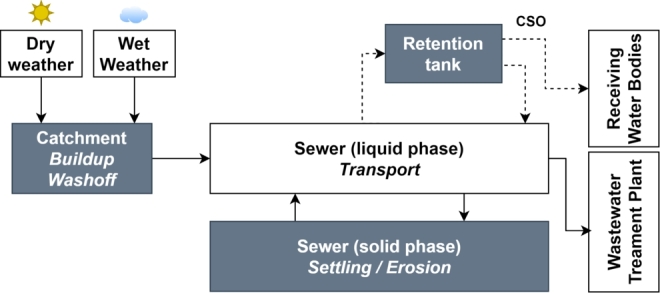
General characterisation of the sewage system [79].

The sewage system infrastructure is designed to cope with the inflow of wastewater originating from the daily use of water discarded from households and industry, which is defined as the Dry Weather (DW) inflow. However, during rain events, a secondary inflow enters the sewer system, also known as Wet Weather (WW) inflow, and the capacity to store the volume of water might exceed the maximum storage volume of a retention structure (pipe or tank). As can be seen in Fig. 2, this then results in a CSO event.

The composition of the sewage network includes complex features, such as subsystems with nonlinear dynamics with delays such as open-flow channels, pressured pipes, weirs, siphons and flow dividers. Moreover, the control of sewage networks is achieved through a combination of continuous variables (flows and volumes), and discrete variables (on-off control devices and overflow events) [54]. In general, the available storage volume in retention tanks is the main controlled variable in the network, which is manipulated by an actuator (valve or pump) located at the outlet of each retention tank that controls the outflow. Normally, sensors are included for level and outflow measurements, but estimators are often observed in their absence [6].

Sewage has both a liquid and a solid phase and they can be described using parameters such as Total Suspended Solids (TSS), Biochemical Oxygen Demand (BOD), Chemical Oxygen Demand (COD), Nitrates and Nitrites (NOX), Total Kjedahl Nitrogen (TKN), Total Phosphorus (TP) and Dissolved Oxygen (DO) [62] as well as concentration of specific compounds such as ammonia (NH_4_^+^). The chemical and biological process of the sewage system are often viewed as a reactor where all phases interact [84] and solid particles are subjected to dynamic behaviours as described in [71] and [64].

Mathematical descriptions of pollutants and their known behaviour are important to understand these phenomena and improve sewage systems models.

### 2.1 Modelling taxonomy

**Figure 3:**
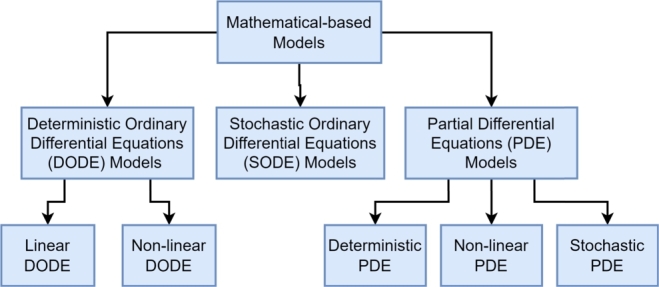
Mathematical-based model Taxonomy by [98].

Several papers developed strategies for modelling of sewage systems. In [98], a comprehensive review of mathematical descriptions of pollutants is presented, which summarises the mathematical-based models as in Fig. 3.

The author details the mathematical modelling methods:

1. Deterministic ordinary differential equations models (DODE)
2. Stochastic ordinary differential equations (SODE)
3. Partial differential equations (PDE)

These methods are used to describe the space-time evolution of pollutants dynamically, and the author presents several approaches, from a simple Linear DODE (LDODE), to the highly complex Stochastic PDE (SPDE). The final remark of the author expresses the need of high computational requirements for the complex methods proposed.

**Figure 4:**
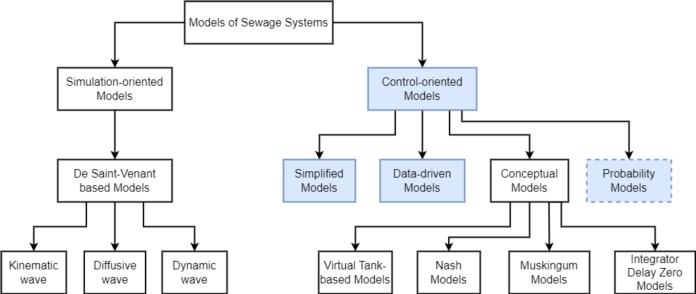
Sewage system model taxonomy proposed by [32].

A more general classification was proposed in [32] based on a literature review, as seen in Fig. 4. This taxonomy is more relevant for this review paper, because it focuses on modelling techniques applied specifically for RTC design and application, considering the time constraints, computational requirements and accepting that RTC systems do not necessarily require highly complex descriptions of the infrastructure. Therefore complex models such as DODE, SODE and PDE are out of the scope of this paper. For further information in these models, the reader is encouraged to read [3], [49], [56], [71] and [98].

The taxonomy in Fig. 4 separates models based on its purpose: simulation-oriented or control-oriented. In this paper the focus is on water quality models needed for control implementation and the analysis of simulation-oriented models is disregarded.

Control-oriented models are specifically designed to be used with control systems, which also needs to factor-in the relationship between accuracy as well as the type of controller applied. They are separated in three main classes:

• **Simplified models** are mathematical formulations of the sewage network with simplified dynamics for the sake of a reduced computational burden. These models are characterised by a set of equations that provide an approximate behaviour of the real system.
• **Data-driven models** use process data and machine learning techniques that provide the output of the system without any knowledge of the behaviour of the sewage network. Data-driven models are commonly known as “black-box” models, since the actual mathematical model is unknown. The main use of data-driven models for control is, therefore, prediction, especially in methods where future predictions are essential for good performance.
• **Conceptual models** describe the system through a parametrisation of the tanks in the network. Each sub-category shown in Fig. 4 represents a different way the tank is parameterised. The conceptual models are commonly used for volume-based control algorithms. Since no related conceptual model was found in the literature this type of model is not further reviewed in this paper.

**Figure 5:**
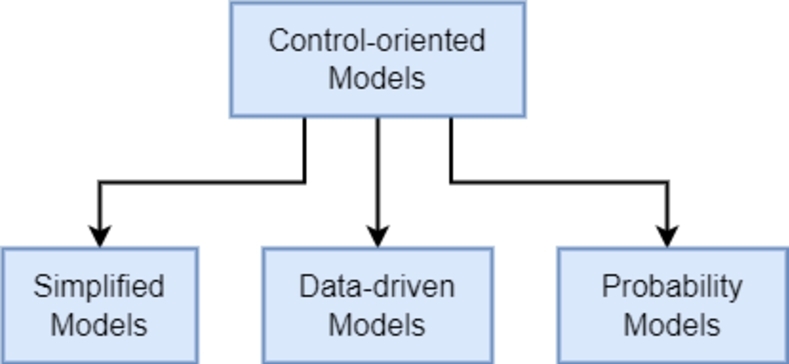
Proposed sewage system control-oriented model taxonomy.

Literature research revealed methods for defining a quality model based on probability [77] that are not contemplated in the current taxonomy and, therefore, a new branch is proposed in this paper as shown in Fig. 5.

• **Probability models** consider that not every variable of the process is deterministic and a solution is to describe the nondeterministic variable with a statistical representation [68].

The literature in pollution-based control showed that current modelling approaches fit the taxonomy proposed. There are several important pollution-based modelling topics, such as sewer corrosion [39] [94] and greenhouse gas emission models [27], however this paper focuses on hydrological models and details different examples of models for each taxonomy.

### 2.2 Simplified models

The complexity of modelling a system using phenomenological non-linear equations might prevent the model to be used for controlling the network, due to high computational cost to calculate the model's output in real time or difficult calibration of the variables to generate a reliable model. In addition, a complex model in general also turns the controller design into a much more complicated task. A solution is an approximation, which can be achieved by linearisation of the non-linear model or by assuming a simplified dynamic behaviour.

In the sewage network, an example of a simplified model is to approximate hydraulic elements, such as valves, pumps, pipes and tanks with simple functions. The dynamics of pumps and valves are commonly simplified due to their fast action compared to the slower sewage network overall dynamic. Pipes and tanks can also be simplified, for example, using a plug-flow method which considers the pollutant flow with constant velocity, volume and concentration through partially filled pipes, defined by a simple time-delay. Similarly, a tank is normally considered as a fully-mixed reactor.

In the next sections simplified models used for pollution-based control and their application are reviewed.

#### 2.2.1 Concentration balance model

[48] defines a quality model for the sewage system, where a simple tank-based model is used with three assumptions for the pollution within the description: (i) one global indicator for pollution, (ii) a simple dilution effect and (iii) homogeneous concentrations in the tank.

**Figure 6:**
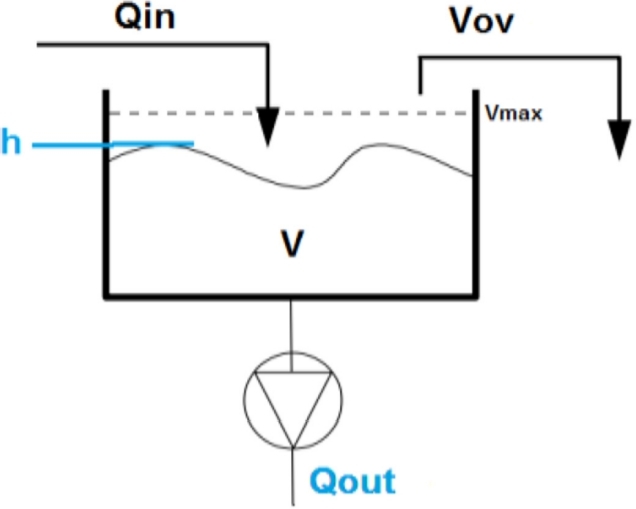
Tank model for wastewater quantity modelling [48].

Mahmoodian describes the sewage system in terms of a simple tank-based quantity model where the outflow of the tank is controlled. Each tank is modelled individually and contains a sensor for measuring the level, as depicted in Fig. 6.

The inflow $Q_{in}$, at time *t*, can be derived either by measurements or using a mass balance equation in a tank by knowing the outflow in the tank, $Q_{out}$, the volume *V* calculated using the level measurement and the volume overflow $V_{ov}$, as seen in Equation (1):

(1) $$V(t)=V(t-\Delta t)+Q_{in}(t)\Delta t-Q_{out}(t)\Delta t-V_{ov}(t)$$

**Figure 7:**
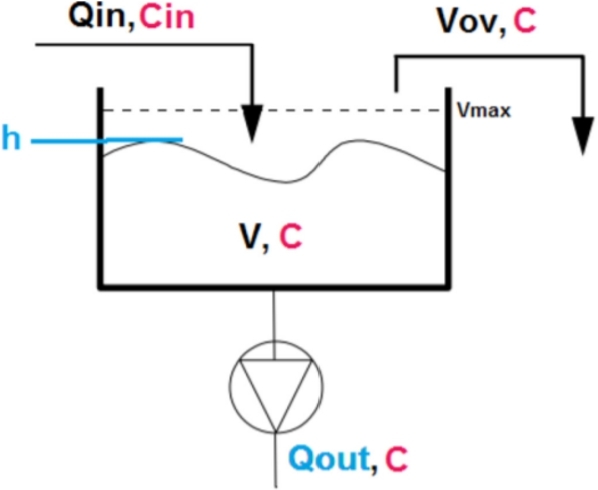
Tank model for wastewater quality modelling [48].

Then, by connecting each tank using plug flow, the network is fully described. This mathematical formulation of the tank model can be extended to consider the concentration of the wastewater entering and leaving the tank, as presented in Fig. 7.

Similarly to volume, the concentration *C* is calculated using the mass balance:

(2) $$m(t)=m(t-\Delta t)+mf_{in}(t)\Delta t-mf_{out}(t)\Delta t-m_{ov}(t)$$

where *m* is the mass and *mf* is the mass flow. By considering m(t)=C(t)V(t), where *C* is the concentration in the tank and $C_{in}$ is the concentration of the inflow, and mf(t)=C(t)Q(t), Equation (2) can be written as Equation (3):

(3) $$C(t)V(t)=C(t-\Delta t)V(t-\Delta t)+C_{in}(t)Q_{in}(t)\Delta t-C(t-\Delta t)Q_{out}(t)\Delta t-C(t-\Delta t)V_{ov}(t)$$

By manipulating Equations (1) and (3), and isolating C(t), the concentration is modelled as Equation (4):

(4) $$C(t)=\frac{C(t-\Delta t)V(t-\Delta t)+C_{in}(t)Q_{in}(t)\Delta t}{V(t-\Delta t)+[Q_{in}-Q_{out}]\Delta t-V_{ov}(t)}-\frac{C(t-\Delta t)Q_{out}(t)\Delta t-C(t-\Delta t)V_{ov}(t)}{V(t-\Delta t)+[Q_{in}(t)-Q_{out}(t)]\Delta t-V_{ov}(t)}$$

The author mentions that this model does not rely on measurements of concentration, which is beneficial for applications since it is not required to change the real network for the purpose of a pollution-based RTC [48]. However, it is necessary to estimate the concentration and, therefore, Mahmoodian proposes the use of a first order Taylor series to estimate $C_{in}$ and calculate the uncertainty propagation from the derived approximation. The Taylor series uncertainty propagation is characterised by a $U_{X}$, which is the standard deviation of X such that variance of *X* is $Var(X)=U_{X}^{2}$. Therefore, the standard deviation for the concentration is given by Equation (5):

(5) $$Var(C(t))=U_{C(t)}^{2}=\sum_{i=1}^{6}{\left(\frac{\partial C(t)}{\partial A_{i}}\right)}^{2}U_{A_{i}}^{2}$$

where $A_{i}$ indicates each variable of the system.

$$A_{1}=C(t-\Delta t),\;A_{2}=V(t-\Delta t),\;A_{3}=Q_{in}(t),A_{4}=C_{in}(t-\Delta t),\;A_{5}=Q_{out}(t),\;A_{6}=V_{ov}(t-\Delta t)$$

Hence, to avoid the need for measurements, the concentration can be approximated using Taylor series and the uncertainty model from Equation (5) results in Equation (6):

(6) $$U_{C(t)}^{2}=U_{C(t-\Delta t)}^{2}{\left(X(t-\Delta t)\right)}^{2}+U_{C_{in}(t-\Delta t)}^{2}{\left(1-X(t-\Delta t)\right)}^{2}$$

where X(t−Δt) is defined in Equation (7)

(7) $$\begin{array}{cc} X(t-\Delta t) & =\frac{V(t-\Delta t)-Q_{out}(t)\Delta t-V_{ov}(t)}{V(t)}\\ & =\frac{V(t)-Q_{in}\Delta t}{V(t)}\\ & =1-\frac{Q_{in}(t)\Delta t}{V(t)} \end{array}$$

Moreover, considering $Q_{in}(t)=Q_{\text{DW}}(t)+Q_{\text{WW}}(t)$, of which $Q_{\text{DW}}$ is the inflow derived from dry weather and $Q_{\text{WW}}$ is the inflow from wet weather, resulting in the uncertainty model in Equation (8):

(8) $$U_{C_{in}(t)}^{2}=U_{C_{\text{DW}}(t)}^{2}{\left(\frac{Q_{\text{DW}}(t)}{Q_{in}(t)}\right)}^{2}+U_{C_{\text{WW}}(t)}^{2}{\left(\frac{Q_{\text{WW}}(t)}{Q_{in}(t)}\right)}^{2}$$

Using Equations (6) and (8) it is possible to calculate the uncertainty of the unmeasured concentration in the tank and use this information for controlling the sewage system with a pollution-based RTC.

This modelling is relatively straightforward, easily incorporated in a optimisation algorithm, however the assumptions of a global concentration indicator overly simplify the model in that it makes no distinction between soluble compounds and particulate matter.

#### 2.2.2 Total suspended solids balance model

**Figure 8:**
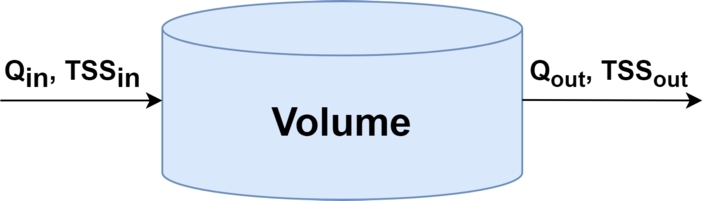
Water tank container [79].

Instead of modelling the concentration using a global indicator, some authors propose the use of specific analytes, such as TSS or COD. This is the case for the model in [79], which initially defines a hydraulic model assuming a water tank container as in Fig. 8.

Sun proposes three different modelling equations for the TSS:

(9) $$\text{Model 1:}\;{\text{TSS}}_{out}(t+1)=(1-c){\text{TSS}}_{out}(t)+c{\text{TSS}}_{in}(t)$$

where TSS_in_ and TSS_out_ are the TSS entering the structure and the TSS leaving the structure, respectively. Equation (9) can be generalised such that the calibration parameter *c* can be independent for TSS_in_ and TSS_out_, showed in Equation (10):

(10) $$\text{Model 2:}\;{\text{TSS}}_{out}(t+1)=c_{1}{\text{TSS}}_{out}(t)+c_{2}{\text{TSS}}_{in}(t)$$

which $c_{1}$ and $c_{2}$ are calibration parameters for TSS_out_ and TSS_in_ respectively. Furthermore, as mentioned previously, the TSS is affected by sedimentation, erosion and time delays in its dynamics. Thus, Sun upgraded the model to add this information and improve the characterisation, resulting in Equation (11a) and (11b):

(11a) $$\text{Model 3:}\;{\text{TSS}}_{out}(t)=c_{vc}{\text{TSS}}_{in}(t-d)+e_{p}$$

(11b) $$Q_{out}(t)=t_{vc}Q_{in}(t-d^{\prime })$$

where *d* is the delay of TSS, $d^{\prime }$ is the delay of the flow and $c_{vc}$, $t_{vc}$ and $e_{p}$ are parameters that need calibration. Sun et al. discusses that, using these modelling methodologies, it is possible to develop a pollution-based RTC, although it is necessary to have an online calibration of several parameters, which are done via a general algebraic modelling system (GAMS) using historic or real-time data from telemetry systems. In a comparison between the three proposed models, all three behaved similarly, whereas Model 2 was slightly better. It is suggested to use a simulation software such as SWMM [69] for the real-time calibration of parameters. For that reason, these models are highly sensitive to changes in the sewer system and need to be actively calibrated to guarantee a reliable prediction.

#### 2.2.3 Effluent quality index model

The last simplified model presented establishes an effluent quality index that is used to measure the pollution load, integrating the following pollutants together: TSS, BOD, COD, NO_x_, TKN and TP. The model characterisation can be seen in [42] and is further described in [62]. It is a unique approach by modelling several types of pollutants simultaneously, allowing a more complex control of the sewage system. The effluent quality index (*EQI*) is defined by Equation (12):

(12) $$EQI=\frac{1}{1000(t_{f}-t_{0})}\int_{t_{0}}^{t_{f}}C_{t}Q_{e}(t)dt$$

where $Q_{e}(t)$ is the overflow rate, $t_{f}$ and $t_{0}$ are the final and initial instants, respectively, and:

$$C_{t}=\beta_{\text{TSS}}C_{\text{TSS}}+\beta_{\text{COD}}C_{\text{COD}}+\beta_{\text{BOD}}C_{\text{BOD}}+\beta_{\text{NOX}}C_{\text{NOX}}+\beta_{\text{TKN}}C_{\text{TKN}}+\beta_{\text{TP}}C_{\text{TP}}$$

where $C_{x}$ and $\beta_{x}$ corresponds to the concentration and weights for each pollutant *x*, respectively.

The quality index modelling increases the complexity of the optimisation by increasing the number of variables, while also requiring measurement (or estimation) of several concentration parameters. This introduces more uncertainties to the optimisation where those measurements are unreliable or nonexistent, degrading the control of the network. However, this technique greatly improves the versatility of the algorithm, allowing to control different types of pollutants independently. The model also enables the control algorithm to be customised depending on the requirements of the network, increasing the importance of certain pollutants for the controller.

#### 2.2.4 Section summary

Simplified models are generally used in sewage system applications which have highly non-linear and complex dynamics, approximating the system with sufficient accuracy for control. Although the assumptions needed for a simplified model might seem excessively simplistic, for real time control, these models are attractive because of their low computational effort and easy implementation.

### 2.3 Data-driven models

The use of data and machine learning techniques is the foundation of data-driven models. When using simplified models, information of the behaviour of the system is lost for the sake of faster computation and, also, the calibration of model parameters is hard to calculate due to uncertainties [4]. Assuming that the model is perfectly calibrated, there is no guarantee that the calibration remains unchanged over time. In fact the system is constantly changing due to shifts in wastewater patterns, seasonal runoff dependencies and land use changes [86]. An alternative is to use data-driven modelling approaches, which rely on real time data processing to represent the dynamics of different variables of the wastewater network, either by using data from software simulation, on field measurements or historical data [83].

The continuous evolution of sensors [8], [14] has led to an increasing number of studies and implementations of data-driven models, encouraging the application in real-time [22]. A few papers can be found that summarise data-driven models for pollution-based systems; [58] reviews detection methods and indicator parameters for quality measurements with an extensive detailing of each type of pollutant's measuring device and a short description of each; [37] reviews and proposes source identification methods for tracking illicit discharges with low cost, showing a good precision for cost-effective analytical techniques; [38] details the modelling of water quality in sewers, providing an extensive review for sewage systems including data-driven models of different purposes.

Data-driven models are easier to adjust to different types of water quality data than other models. The main reason for that is the ability to derive a suitable model regardless of the behaviour of the system through the use of process data and algorithms that mimic human learning. In this section, data-driven models utilised in combination with RTC systems are presented. One notable work is the publication of [1], which studies different deep learning algorithms to predict several water quality parameters. The paper proposed several models used to predict sedimentation and compare the results using five deep learning algorithms: Multi Linear Regression (MLR), Multilayer Perceptron (MLP), Recurrent Neural Network (RNN), Long Short-term Memory (LSTM) and Recurrent Gate Unit (GRU).

#### 2.3.1 Multiple linear regression and neural network techniques for sediment prediction

[1] studied and compared two different data-driven models, a MLR based on [2] and a neural network model based on [21], where both methods are used for predicting in-sewer location and the sediment deposition. It presents an assessment of these models to find the sediment accumulation, where the models inputs and outputs are shown in Table 1, given that the sediment depth, $d_{s}$, is the dependant variable.

**Table 1:** Variables and their definitions [1].

Variable	Definition
*d*_*s*_	Avg. sediment depth in a sewer (*cm*)
NOC	Number of connections to a sewer
D	Diameter of the sewer
Q	Sewer capacity
S	Sewer gradient (%)
*q*_*peak*_	Peak flow (*m*^3^/*s*)
L	Length of the sewer (*m*)
*V*_*peak*_	Velocity that occur at peak flow (*m*/*s*)
*L*_*cum*._	Cumulative sewer length (*m*)
*d*_*peak*_	Depth of flow at peak flow (*m*)
*L*_*dir*._	Cumulative sewer length (*m*)
*τ*_*peak*_	Average tractive stress at peak flow (*pa*)

**Figure 9:**
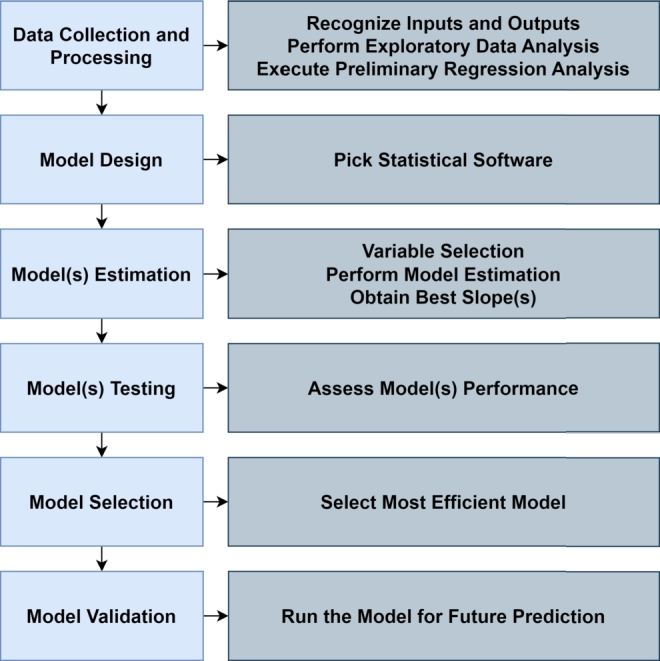
Methodology for MLR and ANN methods [1].

The methodology for both methods is similar and is generalised as shown in Fig. 9. First, the data collection and processing is relevant to determine the variables of the process and perform preliminary analysis of the data. This processing will help to improve the quality of the data and facilitate the use of the data set. The next step is the design of the model, that comprises the selection of several methods that will use the data and create models. In sequence, the estimation and assessment of each model is done and a data set is generated specifically for testing. Finally, a model is selected and validated, defining the most efficient model and using different scenarios for the validation.

#### 2.3.2 Multiple linear regression model

The Linear Regression method is a technique where a single output variable is derived using a simple linear equation from a single input. A more generalised formulation, called Multiple Linear Regression (MLR), approximates the output by multiple inputs simultaneously. The output of the regression model, *Y*, is calculated by using the following model for *n* points collected [43]:

(13) $$Y_{i}=\beta_{0}+\beta_{1}X_{i1}+\beta_{2}X_{i2}+\beta_{p}X_{ip}+\varepsilon_{i},\;\;\;i=1,2,...,n$$

where $\beta_{0}$ is an offset variable, $\beta_{1}$, $\beta_{2}$ and $\beta_{p}$ are the regression coefficients for the independent variables $X_{1}$, $X_{2}$ and $X_{p}$, respectively and *ε* is the error associated with the model. In order to calculate the coefficients *X*, the model in Equation (13) is rewritten to be represented in matrix form, which yields Equation (14):

(14) Y=Xβ+ε

Herein, the output *Y* and error *ε* are *n*-dimensional vectors, the input matrix *X* is n×(p+1) and the coefficient vector *β* is (p+1)-dimensional. Using a least-square regression, the coefficient *β* can be found by Equation (15):

(15) $$\beta ={\left(X^{T}X\right)}^{-1}X^{T}$$

Al-Ani implemented the algorithm for a specific process in Baghdad where a sediment survey was carried out in the sewer system of the city. The implementation of the method was done using the software SPSS Neural Network [15] developed by IBM, which resulted in Equation (16), a linear model using the MLR algorithm as below:

(16) $$d_{s}=0.036L_{cum.}+0.203L+6.685NOC-1.142Z-0.022L_{dir.}-48.493$$

By using collected data, the MLR model is able to predict sediment accumulation throughout the sewer, using a simple approach with very low computational cost. An advantage of this method is that little knowledge of the behaviour of the sewer is required and as long as new data-sets are acquired, the models coefficients are easily re-identified to improve the performance, since it is known that the dynamics of a sewage network change over time. However, the main issue with this approach is the reliability of the data used for the identification and also the error inherent to measuring systems, which increases the error of the MLR method if the sensors used in the data collection are not correctly placed or calibrated.

#### 2.3.3 Artificial neural network model

Modelling sewage systems with Artificial Neural Network (ANN) is a method often seen in the literature. It is mainly applied for regression and classification [36]. The ANN emulates the human brain using underlying relationships between data sets, using an architecture comprised by a Input Layer, a number of Hidden Layers and an Output Layer [1]. The most common architecture is the Multilayer Perceptron (MLP), a feedforward architecture which uses backpropagation as the learning algorithm.

The data used in the MLP approach is divided into three sets: a training set which is used to find the weights of the neural network, the testing set, responsible for checking the error and avoid over-training of the network and the validation set that is used for validation of the model derived from previous training.

The MLP-ANN proposed by Al-Ani et al. was identified using the tool SPSS Neural Network and the resulting model for the same sewer in Baghdad resulted in one neuron with a single hidden layer, while the weights of the network were optimised using a gradient descent method as criteria for training the neural network model. Equation (17) is the final result in a mathematical expression:

(17) $$d_{s}=1.716⁎tanh(X)+0.04$$

where *X* is defined in Equation (18) below:

(18) $$\begin{array}{cc} X= & 0.024+0.114NOC+0.027L-0.262S\\ & +0.758D-1.495q_{peak}+0.207V_{peak}\\ & +0.326Q+0.304q_{peak}/Q\\ & +0.325d_{peak}/D+0.148\tau_{peak} \end{array}$$

There are several advantages of using a ANN-MLP, but, specifically for a sediment accumulation model, the ability to adapt to complex relationships between dependant and independent variables and to changes that occur in the process is very unique [11]. Besides that, there are many open-source software packages available for implementing ANNś, such as scikit-learn, a simple library of machine learning methods using python [59] and OpenNN, a neural network library for C++ [50]. The main disadvantage of using neural networks is finding the best network structure, i.e. the selection of number of layers and number of neurons in each layer, as well as the selection of other parameters of the neural network, which in most cases is done by trial and error although there are optimisation techniques that solve this issue. Another disadvantage is the lack of intuition on the model, since it is difficult to understand the reasoning behind the solution provided by the network.

#### 2.3.4 Deep learning algorithm for multi-source data fusion to predict water quality of urban sewer networks

A similar solution developed in the data-driven modelling framework was proposed by [40], which also used a similar approach to the MLP proposed by Al-Ani, however with the objective of using multi-source data fusion and deep machine learning models to predict several pollutants, such as BOD, COD, NH_4_^+^, TN, and TP.

**Figure 10:**
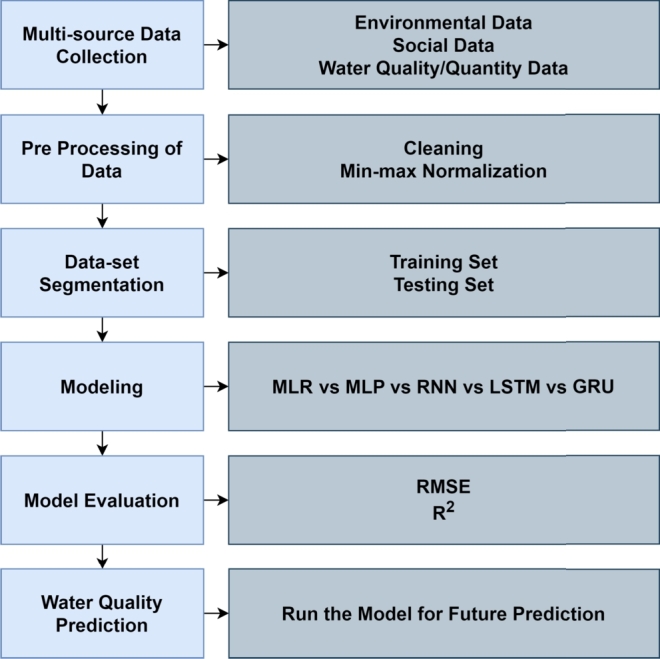
Framework for water quality prediction [38].

Jiang defines the framework as shown in Fig. 10. The first step starts with data collection from several sources, such as (1) environmental data, (2) social data, (3) water quality data and (4) water quantity data. Then, the multi-source data is pre-processed and separated in a training and testing set. Finally, the author compares several deep learning algorithm to predict the water quality. The algorithms the author studied are MLR, ANN-MLP, Recurrent Neural Networks (RNN), Long Short-Term Memory (LSTM) and Gated Recurrent Unit (GRU).

The case study was realised using data from a southern city in China. The input dataset consists of: land area, drain pipe diameter, population, drinking water supply, flow, volume, level, pH, temperature, conductivity and suspended solids. The output indicators are BOD, COD, NH_4_^+^, TN, and TP.

The comparison of the models was done using Root Mean Squared Error (RMSE) and R^2^ metrics. The conclusions are the following:

1. MLP is more suitable for water quality prediction than MLR because traditional machine learning algorithms are better for multi-source data than linear methods.
2. RNN-based methods are better than MLP because those methods have an advantage when the data is time series data.
3. Among the deep learning methods, GRU showed a significant better performance than RNN and LSTM for most indicators.

The author concludes that, although the prediction of water quality using deep learning algorithms has great potential, these models depend on the input indicator selected from the data set used and it is crucial to understand which of these indicators are key for training-learning models.

#### 2.3.5 Section summary

The exponential improvements in computer performance and artificial intelligence increases potential practical implementation of deep learning models with big data, therefore the research on water quality prediction with deep learning has a potential to be key for the future. The ability to adapt to complex dynamics without complete knowledge of such phenomena is appealing but has its limitations.

### 2.4 Probability models

The use of simplified and data-driven models introduces errors due to uncertainties, which means that the prediction using these types of model for controlling purposes might be less reliable. An alternative modelling technique is using models that factor these uncertainties using probabilistic assumptions of the system, also known as stochastic models [68]. The difference between the deterministic and stochastic model is that the later considers the description of the uncertainty within its formulation using a statistical approach.

#### 2.4.1 DO and BOD transport model

**Figure 11:**
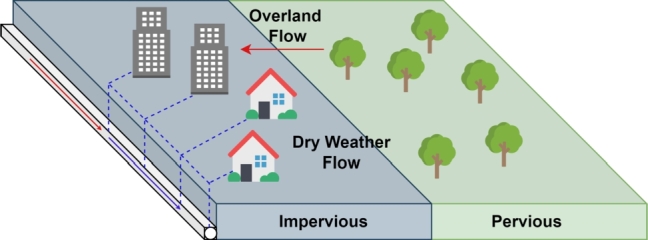
Representation of a catchment by [52].

Water quality indicators such as DO and BOD have a big impact on receiving bodies of water when there is wastewater overflow. Their transport in sewer systems is a complex process and is usually approached in modelling systems by assuming a fixed concentration or by estimation using isolated measurements [90]. However, that leads to the effect that information regarding the concentration during storm events are lost. The solution proposed by [52] is to use a probabilistic model that is able to describe the dynamics of the process. This reduces uncertainties because it does not require a complex parametrisation of the complete sewer network and the model only needs an input value for each catchment. The catchment is shown in Fig. 11, where the overall flow is the combination of a dry weather flow and an overland flow, which is separated as a pervious and impervious overland flow.

Firstly, Morales defines the flow path using state variables, i.e., the pervious overland state ($x_{o_{i},\text{perv}}$), impervious overland state ($x_{o_{i},\text{imp}}$) and the dry weather flow state ($x_{{\text{DWF}}_{i}}$), as well as a series of conduit states ($x_{c}$), where i=1,2,...,n and *n* is the number of catchments. Then, in Equation (19) is shown the probability of the load in each flow:

(19) $$\begin{array}{cc} & P_{x_{o_{i},\text{perv}}}(t)=\frac{\xi_{i,\text{perv}}(t)}{\xi_{T}(t)}\\ & P_{x_{o_{i},\text{imp}}}(t)=\frac{\xi_{i,\text{imp}}(t)}{\xi_{T}(t)}\\ & P_{x_{{\text{DWF}}_{i}}}(t)=\frac{\xi_{{\text{DWF}}_{i}}(t)}{\xi_{T}(t)} \end{array}$$

where $\xi_{i,\text{perv}}$, $\xi_{i,\text{imp}}$ and $\xi_{{\text{DWF}}_{i}}$ are the total constituent loads from the *i*th pervious, impervious and dry weather flow regions at time *t*, respectively.

Morales then describe a travel time probability distribution $f(t^{\prime })$, where $t^{\prime }$ is a random variable associated with the travel time of a drop of water in the network, and where $f(t^{\prime })$ allows to derive the concentration in the outlet, by propagating the concentration through the network. The calculation of $f(t^{\prime })$ extends the work of [70] by taking into account non-conservation of mass in the BOD and DO during the travel time. Finally, the combined pollutograph at the outlet of the sewer network is calculated by convolving the travel time probability distribution and the total load, given by Equation (20):

(20) $$\xi (t)=\sum_{t^{\prime }=1}^{\infty }[f(t,t^{\prime })\times \xi_{T}(t)]$$

The model proposed proved to be an alternative formulation for prediction of BOD and DO in control systems. When comparing with the benchmark software SWMM, the performance of the model showed a good accuracy while requiring less information and time for the implementation. However, this method is much less intuitive and the use of probabilistic variables is not a straightforward approach as the estimation of such variables are complex and harder to grasp.

#### 2.4.2 Bayesian multi-objective calibration for TSS predictions

The representation proposed by [76] is a stochastic model for TSS prediction where the goal is to improve the model errors by using statistical description of the bias. It uses an auto-regressive error model to provide more reliable estimation of TSS and reduce the uncertainty of TSS using a Bayesian multi-objective approach to improve calibration of the model. The general mathematical description of a stochastic model is given by Equation (21):

(21) $$\overset{˜}{Y}(X,\theta^{M},\theta^{\epsilon })=Y_{M}(X,\theta^{M})+{\overset{˜}{B}}_{M}(X,\theta^{\epsilon })+\overset{˜}{E}(\theta^{\epsilon })$$

where $Y_{M}(X,\theta^{M})$ is the deterministic output of the model, $\overset{˜}{Y}(X,\theta^{M},\theta^{\epsilon })$ is the real output of the model, i.e., the sum of the deterministic model with the error, hence $\overset{˜}{Y}(X,\theta^{M},\theta^{\epsilon })$ is a random variable. The inputs of the system are ***X***, the parameter of the deterministic model is represented by $\theta^{M}$ and the parameter of the error is $\theta^{\epsilon }$. The variable ${\overset{˜}{B}}_{M}(X,\theta^{\epsilon })$ is used to describe the model bias and $\overset{˜}{E}(\theta^{\epsilon })$ describe the random measurement noise.

The output $\overset{˜}{Y}$ is modelled as a random variable and the author defined the prediction as a predictive probability distribution $p(\overset{˜}{Y}|X)$, which is calculated using a Bayesian approach:

(22) $$p(\overset{˜}{Y}|X)=\int p(Y^{0}|\theta ,X)p(\theta )d\theta$$

where p(θ) represents the prior knowledge of the parameters ***θ***, $p(Y^{0}|\theta ,X)$ is the likelihood function of the model that measures the probability an output $Y^{0}$ is generated by the model given an input ***X*** and the parameter chosen randomly from p(θ).

Using Equation (22), the model proposed by Sikorska to predict the TSS can be derived, resulting in Equation (23):

(23) $${\overset{˜}{C}}_{TSS}(P^{0},\theta )=C_{TSS}(P^{0},\theta )+{\overset{˜}{B}}_{M_{BW}\times M_{RR}}(P^{0},\theta )+{\overset{˜}{E}}_{TSS}(\theta )$$

where ${\overset{˜}{B}}_{M_{BW}\times M_{RR}}$ comprises the systematic prediction errors of the concentration of TSS, $C_{TSS}$, due to build-up/wash-off (BW) and rainfall/runoff (RR) model components and ${\overset{˜}{E}}_{TSS}$ describes the random errors of the prediction of TSS. Likelihood function of the TSS model is given by $p(C_{TSS}^{0}|\theta ,P_{0})$.

The author further discusses the model by including multiple outputs, such as flows and precipitation. This modelling approach showed an improvement in the prediction efficiency of the TSS model and the bias description led to a more reliable uncertainty estimation. The main challenge of the approach is formulating the model bias using prior information, since the bias is defined to compensate different error sources, which not always have a direct physical interpretation.

#### 2.4.3 Section summary

Probability models for quality prediction are an interesting approach to consider uncertainty in the formulation of the control. Such formulation can increase robustness and improve the performance of the controller in scenarios where randomness is known with good accuracy.

## 3 Pollution-based real time control

Generally, the main goal of Pollution-based Real Time Controllers (P-RTC) is to reduce environmental impact of the wastewater network by introducing pollution information in the controller. The difference between different P-RTC approaches is how this information is handled within the formulation of the controller, the type of pollutant controlled and the framework used to describe the pollution. In this paper is proposed the following P-RTC methods classification:

• **Rule-based Real Time Control**: the control algorithm is defined using heuristics. These heuristics are usually in the form of rules that are imposed to the problem and expect a specific behaviour if the rules are followed. A pollution-based approach using this formulation defines several rules applied to the sewage control system, for example, the algorithm checks if the concentration in a given basin is higher than a certain a value and proposes an action depending on the output of this rule (which is generally a ‘yes’ or ‘no’ output).
• **Optimisation-based Real Time Control**: in this type of method, the overall objective is achieved by minimising (or maximising) a function using optimisation methods, which will provide an optimum operation of the system. Each method can have different objective functions depending on the desired behaviour of the system. One example is a P-RTC based on Model Predictive Control (MPC) which is an algorithm that optimises a cost function in order to control the system over a prediction horizon by using a model to predict the behaviour of the system. In the case of a pollution-based scenario, a common objective defined in the optimisation is to minimise the concentration during overflows of tank structures.
• **Reinforcement Learning-based Real Time Control**: through a reinforcement learning (RL) method, an algorithm learns the behaviour of the system by a trial-and-error experience and by rewarding control actions that meet specific goals. This reward based structure will steer the algorithm towards the actions that maximise the output. For example, A RL-based RTC can be developed to improve the water quality by rewarding control actions where the concentration of overflowed wastewater is reduced [10].

### 3.1 Rule-based RTC

Due to high complexity of the dynamics of sewage systems, the definition of a model is a difficult task to be achieved. Therefore, efforts have been done to create real time control systems which avoided the use of models and one example are rule-based systems, where the control strategy is based on heuristics [96]. Control algorithms based on heuristics are usually more intuitive for operators since the control rules are based on logical statements using expert knowledge of the behaviour of the sewage system. Also, rule-based RTC systems do not require the definition of an optimisation problem and, therefore, the control is less complex and demand less computational power for computing the solution. Combining all these advantages it is clear that rule-based RTC is an attractive solution for controlling sewage systems and several researches focused their effort specifically in designing rule-based RTC systems in an pollution-based framework [96].

#### 3.1.1 Water quality data-informed real-time control

The algorithm proposed by [72] adds a real time controller to the normal operation of the sewage system by adding rules to the behaviour of the system and using the available data. The author defines this controller as a water quality-informed real-time active control due to the fact that the algorithm uses quality (and rain) monitoring to derive a RTC based on rules that are applied and are actively controlling the sewage system.

The author defines four possible control rules in his study, a passive control, a retention control, an on/off control and a TSS control, as can be seen in Table 2. Each of these rules have a different impact on the behaviour depending on the current state of the system and the different variables of the sewage system. The baseline operation is the passive control that is an operating mode where the actuator (a valve) is constantly open.

**Table 2:** Control rules implemented in [72].

Type	Description
Passive Control	Valve always open
retention Control	**Table:** If an event occurs, valve opening = 0% Else, valve opening = 100%
On/off Control	**Table:** if *h* < *h*_*c*_, valve opening = 0% if *h* ≥ *h*_*c*_, valve opening = 100%
TSS Control	**Table:** if *C* ≥ *C*_*c*_, valve opening = 0% if *C* < *C*_*c*_, valve opening = 100%

The retention control mode differs by monitoring inflow events and acting whenever a event occurs, which activates the retention controller and completely closes the valve. The valve is kept closed for a specific duration $t_{d}$, called the retention time, with the objective of storing the inflow volume into the structure. The third mode, on-off control, defines a threshold value for the level of the tank structure, called $h_{c}$, and if the water level is below $h_{c}$, the valve is remained closed, however when it reaches the threshold, is completely opened to drain the volume within the tank.

These two modes are traditional volume-based algorithms, thus, to improve the quality of the sewage, Sharior proposes a fourth mode that takes into account the concentration of TSS inside the tank, called the TSS Control mode. The general idea is similar to on-off control mode, the difference is the threshold refers to the TSS concentration, called $C_{c}$, and the logic of the on-off actuation is different. In the TSS control, when the concentration of TSS is above a threshold concentration, the controller is fully closed, otherwise, the valve is opened. The goal of this procedure is to increase dilution when rain events occur to decrease the concentration and when the concentration reaches a specified value, the valve is opened and drains the tank, sending this volume towards the treatment plant.

The method proposed does not use an explicit model, the author clarifies that measurements were used for the formulation, implementation and simulation of the algorithm.

#### 3.1.2 Quality control based on retention time

An interesting approach for controlling a urban drainage system using a rule-based algorithm was presented by [74], differing from the previous algorithm by using only a volume-based set of rules to influence the quality of the wastewater within the drainage system during dry-weather periods. The goal of this algorithm is to control the settling process in the stormwater basin and, as a result, influence the TSS retention time using a generic formulation that considers a required volume in the basin and information regarding runoff and rainfall [74].

This algorithm is activated during dry-weather periods, whereas during wet-weather, a volume-based optimisation is implemented without any quality aspects. Therefore, since this review paper is only interested in the RTC systems that are directly impacting the pollution in the receiving water bodies, here, the only aspect discussed is the implementation of the, dry-weather, rule-based controller. The author defines four rules to control the wastewater system, as showed in Equation (24) below:

(24) $$\begin{array}{cc} & \text{if}\;\;\;\;t_{nextrain}\leq t_{e}:\\ & \;⟶Q_{t}=Q_{max}\\ & \text{if}\;\;\;\;t_{e}<t_{nextrain}\leq t_{e}+20h:\\ & \;⟶Q_{t}=Q_{max}⁎\frac{t_{e}}{t_{nextrain}-t_{f}}\\ & \text{if}\;\;\;\;t_{e}+20h\leq t_{nextrain}<40h+t_{e}^{max}:\\ & \;⟶Q_{t}=Q_{max}⁎\frac{t_{e}+20h}{t_{nextrain}-t_{f}}\\ & \text{if}\;\;\;\;t_{nextrain}\geq 40h+t_{e}^{max}:\\ & \;⟶\left\{ \begin{array}{cc} & Q_{t}=0\;\;\;\;\;\;\;\;\;\;\;\;\;\;\;\;\;\;\;\;\;\;\;\;\;\;\;\;\;\;\;\;\;\;\;\;\forall t<40h\\ & Q_{t}=Q_{max}⁎\frac{t_{e}}{t_{e}^{max}}\;\;\forall t\in (40h,40h+t_{e}^{max}) \end{array}\right. \end{array}$$

with $t_{e}$ being the emptying time of the basin until availability of the storage volume $V_{req}$ at maximum outflow $Q_{max}(s)$. The author assumes that the main characteristics of the sewage systems are known, such as the inflow to the basin, and therefore no model for control is used.

An important advantage of using this method, as mentioned by the author, is that the use of this set of rules ensures that the available volume within the basin is sufficient to handle the predicted upcoming rain event without overflow, which reduces the discharge of highly concentrated volumes. However, the main disadvantage with this approach is that the control is considered only on a local scale, the downstream areas are not considered globally. Therefore, this method is unable to find a global optimum operation for the sewer system as a whole, which would greatly increase the complexity of the overall method and new rules would need to be applied, whereas the intuitiveness commonly found in rule-based algorithms is lost due to a higher number of rules necessary.

#### 3.1.3 Double-gate storage scheme based on water quality

The next rule-based RTC described was developed by [92], called a double-gate storage scheme for water quality control. The author mentions that traditional interception-storage tank control designs are unable to deal precisely with the pollution problem in combined sewer overflows, therefore, they developed a method which directly influences the variance of a pollutant with the flow-rate. In the paper Wei presents the solution applied specifically for controlling the COD concentration, defining rules in a double-gate scheme. The COD concentration is monitored in the system's storage tank and well chamber and, using this measurement, the rule-based controller will act accordingly.

The well chamber has an intercepting weir that, when opened, directs the inflow directly to a receiving body of water. Also, the chamber is connected through a intercepting pipe to the storage tank which will store the stormwater. During dry-weather, the double-gate is offline, the intercepting weir is closed and the inflow is directed to the municipal sewage pipe. However, when rain events occur, the controller comes online and starts its operation depending on the current COD concentration measured.

• Firstly, a COD concentration threshold $C_{t}$ is defined for the concentration within the well chamber, which indicates a value for the COD that is safe to discharge.
• If the concentration within the well chamber is below the $C_{t}$, this flow is directed to the receiving body water and safely discharged.
• However, when the current COD measurement is above $C_{t}$, the waste intercepting pipe is opened and the waste is stored in the tank while there is enough capacity.
• Eventually the waste in the storage tank is directed to the municipal sewage pipe.

The method developed by Wei showed a great reduction in the concentration of COD in the discharge and also a great improvement over traditional schemes commonly used for dealing with storm-water overflows. The method uses measuring devices to act directly on the concentration, which is a promising technology but which is still complex and expensive for in-situ and on-site quality assessment [19]. The tuning of this method is relatively simple, needing only the threshold COD, but similarly to the previous method, is limited to local storage control and does not provide a globally optimum solution.

#### 3.1.4 Section summary

In Table 3 several papers are shown with more implementations of rule-based RTC. They are separated in Local methods, indicating that each CSO is controlled individually and Local Pollution Rule-based + Global Volume-based Optimisation, that differ by integrating the pollution rule-based local RTC with a volume-based optimisation. In general, rule-based methods are simple and show improvements in local structures regarding load management. However, the capability of globally observing the network system is limited and to design a global rule-based approach requires complex interactions of rules. In the papers reviewed, water level and quality sensors are included in the formulation of the control algorithm, instead, a simplified or data-driven model could overcome situations where measurements are not available.

**Table 3:** Papers on rule-based RTC methods with pollution aspects.

Authors	Year	Type of Control	Description	Type of Pollutant
[93]	1997	Local Pollution Rule-based + Global Volume-based Optimisation	Minimisation of overflow concentration and tracking of pollution discharge to WWTP using a global volume-based optimisation and rule-based local concentration-based control.	Multiple pollutants
[97]	1999	Global	Fuzzy logic control and genetic algorithms are applied to achieve improved pump operations in a combined sewer. It is concluded that current pump operations can be improved by adding the water quality to the input variables.	Global indicator
[87]	2005	Global	Rule-based control of WWTP flow overload using measurement of ammonia in the river.	NH4
[29]	2005	Global	A rule interpreter was developed to make control decisions based on rules (if–then relations), which are evaluated with the aid of fuzzy logic and input values such as water levels, flows, etc.	COD
[35]	2011	Local	A real-time control strategy for separation of highly polluted storm water based on UV–Vis online measurements	TSS using real-time water quality measurement
[30]	2013	Local	Rule-based retention control improving water quality by maximising the retention time of water in the pond in order to increase the TSS removal efficiency through sedimentation.	TSS
[75]	2021	Local Pollution Rule-based + Global Volume-based Optimisation	Rule-based combined with a predictive dynamic control for optimal operation of stormwater system regulators during rainfall periods, while applying the heuristic control for water quality control to detain runoff in the basin during dry weather periods.	Sedimentation

### 3.2 Optimisation-based RTC

Optimisation-based control is a method that relies on an optimisation problem which determines an optimum behaviour based on control objectives through a minimisation (or maximisation) [32]. The advantage is the possibility to control the system globally and the additional use of constraints, which restrict the optimum space solution to guarantee that the control will operate within physical requirements (e.g., a valve actuator will not open more than 100%). The difference between types of optimisation-based RTC is the method used to solve the optimum problem, which might demand a non-linear algorithm, for example, or bayesian optimisation which relies on black-box models. The objectives defined for each method may also change, depending on the desired behaviour.

The optimisation-based RTC can be generalised in state-space representation as Equation (25) [47]:

(25) $$\begin{array}{cc} & \min_{u(n)}J(n)\\ & \text{s.t.}\\ & h(x(n),u(n),w(n))\geq 0\\ & g(x(n),u(n),w(n))=0\\ & x_{min}\leq x(n)\leq x_{max}\\ & u_{min}\leq u(n)\leq u_{max} \end{array}$$

where *x*, *u* and *w* are the states, controlled variables and disturbances, respectively, *J* is the cost function, *h* and *g* are functions that represents the inequality and equality equations, respectively, used in the constraints.

Optimisation-based P-RTC methods, the difference is key to understand the control possibilities. The main contribution is the objective function, which will determine the overall control problem and how the sewage network will behave. The objective might be to reduce overflow. Pollution-based methods are distinct for the use of pollution information, therefore, the objectives of the optimisation problem are extended or changed to deal with water quality aspects, such as minimise load overflow or maximise the load inflow to the WWTP.

#### 3.2.1 Pollution-based MPC for TSS

Policies and regulations on water quality have been made by individual environmental agencies which increase the necessity for specific pollutants to be tighter controlled and monitored. Therefore, new strategies are being studied with the objective of developing RTC systems that are capable of controlling specific pollutants in sewers. In this context, [80] proposed a MPC strategy which seeks to minimise the total amount of pollutants released to receiving bodies of water, focusing on the total suspended solids (TSS), with the justification that this pollutant can be easily measured continuously online.

The pollution-based objective defined by Sun is given by the following function in Equation (26):

(26) $$J=\sum_{i=n}^{n+H_{p}}\lambda \phi_{1}(i)+\beta \phi_{2}(i)+\alpha \phi_{3}(i)+\gamma \phi_{4}(i)$$

$\phi_{1}$, in Equation (27), is an objective used for minimising the combined sewer overflow (CSO) in the sewage system:

(27) $$\phi_{1}(n)=\Delta t\sum_{i=1}^{n_{cso}}q_{cso}^{i}{\left(n\right)}^{2}+q_{bypass}{\left(n\right)}^{2}$$

where $n_{cso}$ is the number of CSO-diverting points, $q_{cso}^{i}$ is the overflow from the i−th point and $q_{bypass}$ is the bypassed overflow from the WWTP when the inflow to the treatment plant exceeded capacity.

$\phi_{2}$, in Equation (28), is an objective that tries to keep the flow incoming to the wastewater treatment plant (WWTP), $q_{w}$, close to the maximum allowed inflow to the WWTP, $q_{lim}^{wwtp}$:

(28) $$\phi_{2}(n)={\left(q_{w}(n)-q_{lim}^{wwtp}(n)\right)}^{2}$$

$\phi_{3}$, in Equation (29), is an objective to smooth variations in the output variable by minimising the difference between the current optimum output u(n) with the previous optimum u(n−1):

(29) $$\phi_{3}(n)=\sum_{i=1}^{n_{g}}{\left(u^{i}(n)-u^{i}(n-1)\right)}^{2}$$

where $n_{g}$ is the number of controlled decision variables of the optimisation problem.

Finally, $\phi_{4}$, in Equation (30), is a quality based objective where the load in overflow of TSS is minimised:

(30) $$\phi_{4}(n)=\sum_{i=1}^{n_{cso}}{\text{tss}}_{cso}^{i}(n)q_{cso}^{i}(n)+{\text{tss}}_{bypass}^{i}(n)q_{bypass}^{i}(n)$$

where ${\text{tss}}_{cso}$ is the TSS concentration of the CSO and ${\text{tss}}_{bypassed}$ is the TSS concentration at the WWTP inlet.

The final optimisation problem is then written in a form similar to Equation (25), where the constraints are established by the physical requirements of the urban drainage network and the model used internally by MPC.

The method uses a simplified model as described in Section 2.2.2.

The method proposed by Sun et al. is simple but effective: The addition of an objective to minimise the load during overflow impacted the behaviour of the controller to improve the quality of the water discharged in receiving waters. The complexity of this method will be relative to which model is used to calculate the predicted value for the TSS (a linear or nonlinear model) and will also depend on if the monitoring of the TSS is done via measurement or estimation. The author mentioned that the method reduced the overall overflow compared to a rule-based approach. When compared to a purely volume-based MPC, the pollution-based MPC decreased the concentration of the overflowing wastewater, thus confirming that a load reduction is possible by adding a quality objective. However, it is mentioned that a certain degree of uncertainty is present and should be addressed in the optimisation to improve the control of the system. The next method reviewed suggests a solution to this uncertainty issue.

#### 3.2.2 Pollution-based MPC considering uncertainty propagation

[48] proposed a method for a real time controller using MPC in a pollution-based framework where a global concentration indicator is added to the optimisation problem as a decision variable. Using this information, the RTC implemented is able to directly affect the behaviour of the pollution within the network in an attempt to reduce the concentration of the discharged wastewater. In order to use concentration in the formulation, the model based on volumes and flow is extended to also consider the concentration and, therefore, be able to predict it. Mahmoodian designed the pollution-based MPC as in Equation (31):

(31) $$\text{min}\;J=\text{min}\sum_{i=n}^{n+H_{p}}\lambda \phi_{1}(i)+\beta \phi_{2}(i)+\alpha \phi_{3}(i)+\gamma \phi_{4}(i)+\mu \phi_{5}(i)+\sigma \phi_{6}(i)+\frac{\delta }{\phi_{7}(i)}$$

in which the cost function is represented by *J* and in its formulation, $H_{p}$ defines is the prediction horizon, *n* represents a discrete time variable and where *λ*, *β*, *α*, *γ*, *μ*, *σ* and *δ* are weights for the objectives $\phi_{i}$, for i=1,...,7. Each objective has a unique characteristic that will influence the behaviour of the wastewater system depending on the weights chosen for this objective.

The objective $\phi_{1}$, in Equation (32), is used to homogeneously store the volume within all tank structures in the sewage network.

(32) $$\phi_{1}(n)=\sum_{i=1}^{N}{\left(V_{i}(n)-\frac{V_{i_{max}}}{\sum_{j=1}^{N}V_{j_{max}}}\sum_{k=1}^{N}V_{k}(n)\right)}^{2}$$

where *V* is the volume in the tank, $V_{max}$ the maximum volume in the tank, i=1,...,N is the number of tanks in the network and $j=1,...,N_{p}$ the number of pipes.

$\phi_{2}$, in Equation (33), keeps the flow towards the wastewater treatment plant near the operating reference value defined by $y_{ref}$.

(33) $$\phi_{2}(n)={\left(y_{ref}(n)-\sum_{i\in N_{k}^{⁎}}Out_{i}(n-d_{i,k})\right)}^{2}$$

where *Out* is the outflow from each tank, $d_{i,k}$ represents the transport time of the $i^{th}$ tank to the destination tank *k* and $N_{k}^{⁎}$ are all tanks draining in the destination *k*.

$\phi_{3}$, in Equation (34), is used to minimise the overflow, where *NL* is assigned as a negative number to keep the objective linear.

(34) $$\phi_{3}(n)=\sum_{i=1}^{N}{\left(Ov_{i}(n)-NL\right)}^{2}$$

where *Ov* is the overflow volume.

$\phi_{4}$, in Equation (35), is used to minimise the load, e.g., reduce the mass in overflow events.

(35) $$\phi_{4}(n)=\sum_{i=1}^{N}{\left(C_{i}(n)Ov_{i}(n)-NL\right)}^{2}$$

where *C* is the concentration in the tank.

$\phi_{5}$, in Equation (36), is to minimise the uncertainty in the load. This objective is interesting in the case where multiple tanks are overflowing and in this scenario, the controller will prefer to let tanks with less uncertainty overflow.

(36) $$\phi_{5}(n)=\sum_{i=1}^{N}{\left(U_{i}(n)-NL\right)}^{2}$$

where *U* is the uncertainty related to the concentration within the tank.

$\phi_{6}$, in Equation (37), is similar to objective $\phi_{1}$, the controller uses this objective to balance the mass over the sewage system's structures homogeneously.

(37) $$\phi_{6}(n)=\sum_{i=1}^{N}{\left(C_{i}(n)V_{i}(n)-\frac{V_{i_{max}}}{\sum_{j=1}^{N}V_{j_{max}}}\sum_{k=1}^{N}C_{k}(n)V_{k}(n)\right)}^{2}$$

$\phi_{7}$, in Equation (38), is used to maximise the load arriving the wastewater treatment plant.

(38) $$\phi_{7}(n)={\left(\sum_{i\in N_{k}^{⁎}}C_{i}(n-d_{i,k})Out_{i}(n-d_{i,k})\right)}^{2}$$

The final minimisation problem is given by Equation (31) subject to the constraints in Equation (39):

(39) $$\begin{array}{cc} & 0\leq V_{i}(n)\leq V_{i_{max}}\\ & 0\leq Out_{i}(n)\leq Out_{i_{max}}\\ & O_{v}(n)\geq 0\\ & C_{i}(n)\geq 0\\ & U_{i}(n)\geq 0\\ & Q_{pipe_{j}}(n)=\sum_{i\in N_{j}^{⁎}}Out_{k}(n-d_{i,k})\leq Q_{pipe_{jmax}} \end{array}$$

where $Q_{pipe}$ is the flow in a pipe and $Q_{pipe_{max}}$ is the maximum flow in a given pipe.

The method uses a simplified model as described in Section 2.2.1.

The pollution-based MPC reduced the mass overflowed compared to a volume-based approach [48]. This method is advantageous mainly due to the use of uncertainty, which is often disregarded during the development of a RTC system. The advantage is that, when considering uncertainty, it is assumed that there is an error related to the measurement or estimation of concentration, which is likely to occur. Therefore, the method is more robust to errors and performance is improved. However, this increased robustness comes at the cost of higher complexity and computational efforts, coupled with the need of a concentration model that requires a non-linear optimisation algorithm, increasing the complexity even further.

#### 3.2.3 Multi-objective optimisation using genetic algorithms

As mentioned in Section 2, wastewater is composed of numerous pollutants and the quality of the water depends on the combination of these pollutants. The method presented by [62] is a pollution-based dynamic control algorithm for a sewer network using a multi-objective optimisation which considers different pollutants within the formulation of the optimisation problem. The author discusses the possible solutions for solving the multi-objective optimisation and selects a genetic algorithm-based optimisation due to the complexity of the problem.

Genetic algorithms (GA) are an heuristic method for solving optimisation problems [17] by mimicking the search for the optimum through biological evolution. In each time step, the solutions, also known as a population of solutions, are updated. Genetic algorithms have basic features to mimic evolutionary behaviour, such as chromosome representation, fitness selection and biological-inspired operators [41]. The overall optimisation problem is similar to Equation (25), however the objective is separated as in Equation (40):

(40) $$\min J(n)=J_{1}(n)+J_{2}(n)$$

The cost $J_{1}$ is related to the minimisation of the pollutant load discharged from a sewer chamber, as can be seen in Equation (41):

(41) $$J_{1}=\sum_{i=1}^{n_{cso}}EQI_{i}$$

where $n_{cso}$ is the number of CSOs and *EQI* is the effluent quality index given by Equation (12).

The second objective $J_{2}$, shown in Equation (42), is used to minimise the cost of the treatment plant while also reducing operational costs from pumps.

(42) $$J_{2}=C_{T}+C_{P}$$

where $C_{T}$ is the wastewater treatment plant cost and $C_{P}$ is the operational cost of pumps in the sewer system. The calculation of both $C_{T}$ and $C_{P}$ are presented in [63].

The method uses a simplified model as described in Section 2.2.3.

The author concludes that the genetic algorithm with the given objectives is capable of minimising the pollution load while reducing pumping costs, which is an unique approach for real time controllers, most methods are more concerned with volume reduction without actually measuring any costs. The algorithm also produced varied temporally and spatially dynamic control settings, obtained depending on regulatory requirements and the financial and environmental situation of the sewer management agency [62]. The implementation of the proposed method is simple, however the calculation of the wastewater treatment plant and pumping cost is not trivial and might lead to uncertainties. However, it is mentioned in the paper that, although GA methods have a rapid convergence, using a genetic algorithm introduces a high computational cost that scales with the size of the sewage network and more efforts are required for real applications.

#### 3.2.4 Section summary

In Table 4 several papers are shown with more implementations of pollution and optimisation-based RTC. The application of optimisation-based RTC is appealing for the efficient load management of urban drainage system. The high computational cost for solving optimisation problems can be a problem for network with non-linear dynamics and high number of decision variables, although this issue is diminished by advancements in computer performance.

**Table 4:** Papers on optimisation-based RTC methods with pollution aspects.

Authors	Year	Type of Model	Type of Optimisation	Description	Type of Pollutant
[65]	1999	Model-free	MPC + GA	Nonlinear MPC using Genetic Algorithm for calculating optimal solution applied to the sewer system, treatment plant and receiving water with the aim to achieve minimum effects of pollution.	DO/COD
[28]	2008	Simplified model	GA	Multi-objective quality control of urban wastewater system using NSGA-II genetic algorithm. The algorithm evaluates the performance of the system directly with regard to receiving water quality indicators, such as for ammonia, phosphorus and dissolved oxygen.	Multiple pollutants
[66]	2010	Simplified model	MPC + Fuzzy	Investigation on fuzzy decision making for multi-criteria optimisation of sewer networks and sewage treatment works regarding the balance of ecologic and economic criteria.	Global indicator
[90]	2014	Simplified model	MPC	Global water-quality optimisation with dynamic cost functions that aims to reduce the global overflow risk across the catchment. The algorithm uses hydrological models with indirect water quality considerations to improve the system performance by dynamically changing the prioritisation of discharges at different CSO structures.	TSS
[89]	2014	Simplified model	Ant Colony	Optimisation-based method designed to improve pollution load management using an ant colony algorithm. The objective is to empty retention tanks using volume and water quality data and discharge the flow to the WWTP while also avoiding exceeding the maximum capacity of the plant.	Multiple pollutants
[78]	2018	Simplified model	MPC	Model predictive control for TSS minimisation in the framework of cyber-physical systems.	TSS
[85]	2020	Model-free	Market-based	Real-time load-balancing algorithm to control distributed storage assets in the collection system, to improve wet-weather flows and water quality at a receiving point.	TSS
[67]	2021	Simplified model	MPC	Integrated multi-software pollution-based control architecture using model predictive control. It proposes a methodology for a closed-loop application between the RTC module and the wastewater system for a water quality application.	TSS
[88]	2022	Simplified model	GA	Genetic algorithm optimisation with cost functions related to the volume of overflow and the overflow quality index for a sewer network, which indicates a mass of polluting units per day. The study compares five optimal control strategies using these metrics.	Multiple pollutants
[12]	2023	Simplified model	Decentralised MPC + Fuzzy	Distributed model predictive control algorithm including fuzzy negotiation among subsystems and a dynamic set-point generation method, applied to a simulated sewerage network using a hydraulic and water quality model for prediction.	TSS

### 3.3 Reinforcement learning-based RTC

Reinforcement Learning (RL) is a machine learning training method in which the main idea is to mimic the human behaviour of learning through environmental interactions. A RL problem involves a trial-and-error situation where the algorithm learns what to do and derive an action of which the numerical reward from taking this action is maximised. By trying each action, the learner finds the action with most reward and influence later inputs of the system, which is why RL is a closed-loop algorithm [81].

The RL method is composed of an agent that takes actions, known as *a*, to interact with its environment expecting a reward, called *r*. During each learning step, the agent receives the state *s* of the environment and is rewarded depending by the quality of its actions to follow certain objectives, which can be a positive or negative reward [9]. Finally, the objective of the RL is to maximise the return function $G_{t}$ as below in Equation (43):

(43) $$G_{t}=r_{t}+\gamma r_{t+1}+\gamma^{2}r_{t+2}+...=\sum_{k=0}^{\infty }\gamma^{k}r_{t+k}$$

where $r_{t}=r(s_{t},a_{t},s_{t+1})$ and γ∈[0,1] is a discount factor weighting the importance of short-term and long-term reward that depends on the current action $a_{t}$, the current and next states $s_{t}$ and $s_{t+1}$, respectively [81].

Although research in this field is growing significantly for storm-water systems regarding flood mitigation and peak flow reduction, such as the works in [9] and [53], a reinforcement learning RTC with the goal of improving water quality is still in its infancy.

#### 3.3.1 Reinforcement learning-based RTC to improve water quality

The research is a novel method using a pollution-based approach proposed by [10] using a reinforcement learning algorithm for developing the RTC. The objective of the controller is to mitigate flooding and improve water quality using a RL-based RTC, where the environment used for the learning process is executed using the software SWMM [69]. The model in SWMM provides the current states to the RL, which are:

$$s_{t}=\left\{ \begin{array}{cc} & \text{Current depth}\\ & \text{Outflow}\\ & \text{Concentration of TSS}\\ & \text{Current valve positions}\\ & \text{Sum of 24 h rain forecast}\\ & \text{Mean value of the 24 h tide forecast} \end{array}\right.$$

The action space (*A*) of the agent is manipulating the valve to any desired value. The reward (r) is calculated by how well the objectives defined are meet by the agent after taking any action within the action space, where the objectives are flood mitigation and pollutant reduction.

The author based the reinforcement learning algorithm on Deep Deterministic Policy Gradients (DDPG) [44] using a deep feed-forward neural network. The DDPG responsible to calculate the control actions (valve opening) over a continuous space while learning to control the system following the desired objectives maximising the rewards. It is presented a RL-based RTC for a sewage system with 2 sub catchments, called Pond 1 and Pond 2. Three agents were created and trained using the DDPG algorithm with the following reward system:

• Agent 1, $r_{ag1}$, is rewarded for minimising total flooding and keeping target pond depths, therefore it is not used for water quality. The reward function is as follows in Equation (44):
  (44) $$r_{\text{ag1}}=\left\{ \begin{array}{c} -\sum Flooding[system,Pond1⁎1000,Pond2]\;F\geq \delta \\ -(|Pond1_{depth}-\tau_{1}|+|Pond2_{depth}-\tau_{2}|)\;F<\delta \end{array}\right.$$
  where Flooding[system] is the total flooding volume, Flooding[Pond1] is the flooding rate at Pond 1 and Flooding[Pond2] is the reward for having Pond 2 depth above a level that causes upstream flooding. *F* and *δ* are the total rainfall in a 24 h window and the rainfall threshold, respectively. Finally, $\tau_{1}$ and $\tau_{2}$ is the reference value for the depth of Pond 1 and Pond 2, respectively.
• In the Agent 2, $r_{ag2}$, is added water quality rewards, which results in it being rewarded for minimising total flooding, keeping target pond depths and minimising the outflow of TSS from the ponds as in Equation (45):
  (45) $$r_{\text{ag2}}=\left\{ \begin{array}{c} -\sum Flooding[system,Pond1⁎1000,Pond2]+TSS[Valve1,Valve2]\;F\geq \delta \\ -(|Pond1_{depth}-\tau |+|Pond2_{depth}-\tau |)+TSS[Valve1,Valve2]+Flooding[system/35000]\;F<\delta \end{array}\right.$$
  where TSS[Valve1,Valve2] is the total TSS load of the controlled valves.
• Agent 3: is used as balance between Agent 1 and Agent 2. The agent is set by using the weights and memory from the trained neural network of Agent 1 as an initialisation for the reward function $r_{agent2}$ from Agent 2, functioning as a pre-training of Agent 3.

Bowes discusses the performance of the controller comparing the results using a RL-based RTC with each of the agents. The increased complexity of Agent 2 compared to Agent 1, due to the extra control objective for minimising the TSS load, revealed that it is harder for a single agent to effectively learn all control policies. Agent 2 was able to effectively learn a policy for minimising the TSS load, however Agent 1 had a better performance for flood mitigation, which indicates that Agent 2 compromised by increasing the flooding. The pre-training performed by Agent 3 using Agent 1's information into Agent 2's reward function resulted in a balanced performance between agents 1 and 2. [10] mention that correctly weighting the RL is highly important to avoid reward gaming, which happens when agent's actions causes undesired behaviours in the environment.

The method uses a model based on neural networks and, therefore, inherently uses a data-driven modelling technique. The author suggests that actual physical or numerical stormwater models could be incorporated into the neural network, but further evaluation is recommended.

#### 3.3.2 Section summary

As mentioned, the use of RL for pollution-based RTC is a recent development. Mullapudi [53] discusses that there are challenges associated with RL, such as formulating rewards, choosing function approximators and deciding the complexity of the control problem. It is stated that for many fields the control is highly sensitive to the formulation of the reward function and requires both expertise and an element of subjectivity for a proper implementation. Moreover, to learn all possible relationships between rewards and control decisions becomes a complex task with high computational demands. Although the paper's statements are related to stormwater system control, the same conclusions should apply to wastewater network control but may also explain the lack of studies in the field.

## 4 Conclusion

The research in pollution-based real time controllers is evolving and the future for its application in sewer systems seems promising. Pollution-based Real Time Control theoretically has the potential for out-performing volume-based systems due to the possibility to explicitly consider pollution in the formulation of the controller. This creates the opportunity to directly affect the quality through the sewage network. P-RTC is also a cost-effective option for improving receiving water quality, reducing the load of the discharged wastewater and guarantee a better functioning of the wastewater treatment plant. However, many obstacles still remain:

• **Nonlinear dynamics are added to the problem when considering concentration within the control formulation**, which increase the challenge of defining models, increase the complexity of the control system and requiring non-linear optimisation. This results in a higher computational cost to calculate the controlled variable in each sampling interval. Several solutions were introduced, such as using a simplified non-linear model based with mass balance equations or data-driven models that, with enough information about the dynamics in the data, will be able to represent the system consistently;
• **Uncertainties are present from measuring devices and estimation techniques** used for real time monitoring of concentration in the wastewater distribution, which increase errors for calculating control actions. The research on uncertainty for control system methods based on pollution is still limited and it is in need of further investigation to understand the impacts in the performance of the controller. Until there is clarity on this aspect, the performance of a P-RTC cannot be properly assessed.

This review started by detailing several modelling methods with pollution intrinsically in its formulation and for control purposes. There is a lack of consensus in the literature of which modelling technique to use, in general. For prediction, although many models are proposed, researchers are still searching for a benchmark effective model that represents the sewage systems with complex dynamics. Many authors use simple formulations for reducing complexity to use in optimisation techniques, but the main issue is the lack of predictability of some dynamics, such as infiltration and first flush effect. Machine learning techniques are a good alternative for creating models with complex dynamics without actually knowing the behaviour of the network, however these algorithms rely on data, which are directly influenced by uncertainty and might result in erroneous and unwanted behaviours by the model.

The P-RTC methods in general are great for improving water quality management. Integrating pollutant prediction models with P-RTC methods has a potential to improve the efficiency on sewage management and comply with governmental water quality targets.

Regarding the control techniques, the research showed a predominance of rule-based and optimisation-based algorithms, however reinforced learning is a promising subject that just recently started getting visibility though the work of [10]. The most simple approach is the RB-RTC, which is defined by a set of rules that are employed to manipulate the sewage system towards a desired quality behaviour. The simplicity of the method is the main appeal for its use, requiring little to zero intervention in the wastewater network system, while also being intuitive for operators. Although many of the RB-RTC implementations are integrated with a volume-based optimisation, usually they are local-based, which means the controllers do not observe the complete network, only local structures individually. Besides that, understanding the dynamics of the sewage system is essential for setting the rules, any mistake will deviate from the desired behaviour and increasing the number of rules will reduce the intuitiveness of the algorithm.

The optimisation-based RTC literature showed that this category is mainly comprised by methods based on model predictive control and genetic algorithms. The similarities are in the implementation of objective functions that determine the control actions and the use of an optimisation method, however each method solves the problem differently. Optimisation-based methods are, more often than not, global, as opposite to RB-RTC, and the control actions are optimal, i.e., the controller calculates the best optimal action. Nonetheless, these methods are complex and demand, simultaneously, knowledge from operators to properly configure the controller and higher computational cost from hardware. A third alternative reviewed is reinforcement learning-based methods, which are algorithms based on mimicking natural evolution and predicting the behaviour through a learning process. The RL-based RTC is new and more developments are needed to understand the performance compared to other methods, however it showed as a promising non-optimisation dependant alternative for implementing global real time control.

The literature in pollution-based RTC methods focused its efforts in presenting novel controllers and comparing the performance with previous implementations, however there is still much to be researched. For future research topics, the authors suggest:

• An in-depth water quality control process identification study is missing, complementary to the research in [91], which studies and proposes the identification of variables such as the volume, flow rate, velocity and depth, in a pilot sewage network using the conservation of mass and momentum;
• The inflow concentration $C_{in}$ is one of the most important variables for an optimal control of the wastewater network and it is uncertain. Research studying the uncertainty in $C_{in}$ and how to handle it in RTC systems is limited [55]. Robustness is also important for keeping stability, but research in this field is not existing for P-RTC methods;
• There are some studies in stochastic volume-based RTC ([82] and [33]) and drinking water networks ([24] and [18]), however using this framework within pollution-based RTC is presently missing and this could be an interesting research field;
• Data-driven models for prediction in SUDS are a well-known topic in literature and the application of these models in volume-based RTC can be observed in [5], [23] and [45]. However, the research of data-driven methods for water quality prediction used in P-RTC is still in its infancy, thus being an appealing research topic for future research;
• Further developments on reinforcement learning RTC that is based solely on the research done by [10], such as different reward shapes, applying a multi-objective RL P-RTC to deal with both quantity and quality targets or study RL optimisation for different pollutant types;
• More studies in real applications of pollution-based RTC.

The potential of pollution-based RTC to have an impact on water quality may be considered to be illustrated by the increase in research efforts. Also realistically, urban drainage systems are ready for implementation of these innovative methods because they are increasingly outfitted with modern PLC and SCADA systems. Hence, future researchers need to focus on real implementation of pollution-based methods and demonstrate the benefits.

## CRediT authorship contribution statement

**Rodrigo da Silva Gesser:** Writing – original draft, Visualization, Validation, Methodology, Investigation, Formal analysis, Data curation, Conceptualization. **Holger Voos:** Supervision, Resources, Funding acquisition, Formal analysis. **Alex Cornelissen:** Writing – review & editing, Validation, Supervision, Resources, Project administration. **Georges Schutz:** Writing – review & editing, Supervision, Resources, Project administration, Conceptualization.

## Declaration of Competing Interest

The authors declare the following financial interests/personal relationships which may be considered as potential competing interests: Rodrigo da Silva Gesser reports financial support was provided by Fonds National de la Recherche - FNR, grant reference 17139914. Rodrigo da Silva Gesser reports financial support and writing assistance were provided by 10.13039/100008665University of Luxembourg, grant reference 17139914. Georges Schutz reports a relationship with RTC4Water that includes: funding grants and non-financial support.

## Data Availability

No data was used for the research described in the article.
